# Supplementary material for: Minimisation of metabolic networks defines a new functional class of genes
Source: Nat Commun. 2024 Oct 31;15:9076. doi: 10.1038/s41467-024-52816-2 (PMC11528065; doi:10.1038/s41467-024-52816-2)
Supplement: Supplementary file 1 — Supplementary Information [file 41467_2024_52816_MOESM1_ESM.pdf]

Supplementary Information

Minimisation of metabolic networks defines a new functional class of genes

Giorgio Jansen[1,2], Tanda Qi[3], Vito Latora[4,5], Grigoris D. Amoutzias[6], Daniela Delneri[3], Stephen G. Oliver[1*,] Giuseppe Nicosia[1,2*]

[1]Department of Biochemistry, University of Cambridge, UK; [2]Department of Biomedical & Biotechnological Sciences, University of Catania, Italy; [3]Manchester Institute of Biotechnology, University of Manchester, UK; [4]School of Mathematical Sciences, Queen Mary University of London, UK; [5]Department of Physics and I.N.F.N., University of Catania, Italy: [6]Bioinformatics Laboratory, Department of Biochemistry & Biotechnology, University of Thessaly, Greece

*These two authors made equivalent contributions to the research.

This research work includes 8 excel files (8 Supplementary Data):

Legends of the Supplementary Data.

SD1 All Frequencies.xlsx

All results for all organisms studied. This table summarizes all the results in terms of frequency genes in the MMNs found by the algorithm Each sheet corresponds to a single organism's genome scale-model tested Frequency Definition For each organism, excluding S. cerevisiae, the first column represents the frequency in the MMNs found for that model for each of the genes in the second column. So, for example, a frequency of 1 means that the corresponding gene is present in the 100% of the MMNs, a frequency of 0.5 correspond to a gene present in the 50% of MMNs and so on. S. cerevisiae tabs. In these two tabs the first columns describe the genes, while there are more columns for the results, having considered more media. The 'All' columns refers to the frequency considering all the MMNs in all the media tested. The 'All Aerobic' columns refer to the frequency considering all the MMNs in the 3 aerobic media tested The 'All Anaerobic' columns refers to the frequency considering all the MMNs in the 3 anaerobic media tested Then there are the single columns referring to the MMNs found separately by the algorithm for each medium considered.

SD2 mandGenesMagnificentImpactMean.xlsx

We considered different metabolic networks resulting from their deletion and measured the reduced capability to produce precursors for the biomass pseudo-reaction, as defined in the metabolic network (by Flux Variability Analysis) detailed results in Supplementary Data 2. The table describes the simulated impact of the magnificent seven genes and all their possible combinations. For each deletion strain considered (columns) the ability of the metabolic network to grow or produce the maximum possible amount of a biomass' precursors (rows) is tested Global: the first two sheets consider an average of the strain in all the 6 media considered In the 'Perc' sheets are reported the percentual variation from the WT, colouring the strains with a more severe reduction The next tab report the absolute value of fluxes predicted All the next tabs consider a single medium (please consider that there is a duplicate for each tab).

SD3 Functions and Compartments KO.xlsx

Information on the compartments and functions in the various used media.

The table reports the frequency of KO divided by functionality or compartment location Each tab is for the function or compartment KO in the 6 different media. The columns B and C are the number of genes in WT and the relative weight in the genome scale model The columns D and E are similar, but referring to the smallest MMN found by the algorithm. The last two columns are again similar, but with the mean over all the MMNs found.

SD4 comp-datasetev13.xlsx

Comparison with published prediction of dispensable and core essential genes.

The table summarizes the comparison of our results with the study. The genes in the model marked as essential were not considered. In the first tab all the genes also present in the model are reported. The first 6 columns are taken from the reference study. In the columns labelled as 'Simulation (Presence in MMNs)' our results are reported instead. A scale of colour from green (or blue for dispensable) to red is used to highlight the genes with an outcome more or less similar to the result reference study. A last remark is sometimes added in the last column. The next two tabs consider only the subsets of experimental results or the computer prediction of the reference study for the genes that are also present in the model The final tab report all the gene present in the reference study. Most of the genes are not present in the model, hence the data in our simulation for them are left blank.

SD5 transporters_related_genes.xlsx

This supplementary data contains the list of genes considered as transporters in our study, with their SGD description and their category. This supplementary data lists all the transporter genes included in the WT metabolic model; it also provides information on their functional categories (e.g., amino acid transport, ammonium transport, ion transport, etc.) as well as the SGD description of their functional role (SGD accessed on 22.06.24).

SD6 Primers.xlsx

All primers used for construction of the deletion cassettes and those used for confirmatory PCRs.

SD7 GR Td Y.xlsx

Growth rate, doubling times, and yields in both SD and YPD for the wild-type strains and the multiple mutants that we constructed.

SD8.xlsx

Statistical test used for comparison in Fig 2: two-sided Wilcoxon Test.

Methods

In vivo validation experiments

Random selection of non-NED genes

For the random selection, none of the data were excluded. Non-*NED* genes were selected with the random initialisation function of Python3 (using random initial seeds) from the list of non-*NED* genes.

Construction of double, triple and quadruple mutants

Multi-gene deletants were constructed by deleting genes of interest sequentially from single-gene deletion mutants (carrying *kan*MX) via multiple rounds of target gene replacement (Wach A. et al. 1994) using either antifungal resistance cassettes (*nat*NT2, *hph*MX6) or a *URA3* marker[40]. Successful transformants were confirmed via colony PCR (Wach A. et al. 1994). All primers used for construction of the deletion cassettes and confirmatory PCRs are listed in Supplementary Table 6.

Supplementary Table 1. Summary of the minimisation results and WT growth rates in the different conditions and GR thresholds for the *S. cerevisiae* model (yeast 8.3.1, PMID 31395883) used in this study. The model includes 1133 genes of which 157 are essential genes. The fourth column reports the number of Minimal Metabolic Networks (*MMN*s) found by the algorithm. Columns 5-7 report the maximum, minimum and average number of KOs respectively. The eighth column reports the number of Network Efficiency Determinants (*NED*s) for each medium. In the penultimate column there are 4 values, the first two values (i.e., 46 and 7) indicate the *NEDs*, respectively for the three aerobic and anaerobic media, when the threshold on the GR is 1%; similarly, the remaining two values (i.e., 38 and 7) indicate *NEDs* when the threshold on the GR is 10%. In the last column there are two values (i.e., 7 and 6) indicating the *NEDs* for the 6 media, with the GR threshold at 1% and 10% respectively.

<table>
<tr><th>Threshold</th><th>Medium</th><th>GR of WT (h^{-1})</th><th>#MMNs</th><th>max KOs</th><th>min KOs</th><th>mean KOs</th><th>NED Genes</th><th>NED Genes per Aerobic and Anaerobic conditions and Thresholds</th><th>NED Genes for the given threshold</th></tr>
<tr><td rowspan="6">1%</td><td>SD</td><td>0.47*</td><td>787</td><td>818</td><td>789</td><td>806.89</td><td>81</td><td rowspan="3">46</td><td rowspan="6">7</td></tr>
<tr><td>Minimal</td><td>0.30*</td><td>756</td><td>782</td><td>761</td><td>819.53</td><td>117</td></tr>
<tr><td>YPD</td><td>0.63</td><td>772</td><td>834</td><td>811</td><td>826.41</td><td>65</td></tr>
<tr><td>SD Anaerobic</td><td>0.32</td><td>773</td><td>912</td><td>867</td><td>902.74</td><td>17</td><td rowspan="3">7</td></tr>
<tr><td>Minimal Anaerobic</td><td>0.26</td><td>769</td><td>825</td><td>806</td><td>819.53</td><td>79</td></tr>
<tr><td>YPD Anaerobic</td><td>0.32</td><td>799</td><td>909</td><td>868</td><td>898.56</td><td>20</td></tr>
<tr><td rowspan="6">10%</td><td>SD</td><td>0.47</td><td>792</td><td>836</td><td>782</td><td>817.34</td><td>61</td><td rowspan="3">38</td><td rowspan="6">6</td></tr>
<tr><td>Minimal</td><td>0.30</td><td>791</td><td>789</td><td>762</td><td>778.94</td><td>100</td></tr>
<tr><td>YPD</td><td>0.63</td><td>799</td><td>850</td><td>808</td><td>836.46</td><td>49</td></tr>
<tr><td>SD Anaerobic</td><td>0.32</td><td>778</td><td>912</td><td>868</td><td>903.90</td><td>17</td><td rowspan="3">7</td></tr>
<tr><td>Minimal Anaerobic</td><td>0.26</td><td>801</td><td>832</td><td>800</td><td>824.38</td><td>72</td></tr>
<tr><td>YPD Anaerobic</td><td>0.32</td><td>791</td><td>909</td><td>857</td><td>899.34</td><td>20</td></tr>
</table>

*For these two media aerobic simulations, the oxygen uptake rate was constrained to 2 mmol/(h*gDW), otherwise the growth rates became infeasibly high (1.87 and 1.16 h^{-1}, respectively). The *in vivo* values for the WT aerobic growth rates that we obtained in our liquid culture experiments were 0.44 h^{-1} for SD and 0.63 h^{-1} for YPD (see Supplementary Data 6).

Supplementary Table 2. The *NEDs* for all the *MMNs* of *S. cerevisiae* in the 6 different external conditions. When considering the 1% maximum reduction of Growth Rate, only 7 genes are always present in the *MMN*s regardless of the simulated medium considered.

Systematic Names	Standard Gene Names	Name Description	Frequency (1% Biomass Threshold)	Frequency (10% Biomass Threshold)
YBR126C	*TPS1*	Trehalose-6-Phosphate Synthase	1	1
YDR074W	*TPS2*	Trehalose-6-Phosphate Synthase/phosphatase	1	1
YER026C	*CHO1*	CHOline requiring	1	1
YGR204W	*ADE3*	ADEnine requiring	1	1
YKL067W	*YNK1*	Yeast Nucleoside diphosphate Kinase	1	1
YKR067W	*GPT2*	Glycerol-3-Phosphate acylTransferase	1	1
YMR205C	*PFK2*	PhosphoFructoKinase	1	0.87731
YLR058C	*SHM2*	Serine HydroxyMethyltransferase	0.87393	0.78704
YDR127W	*ARO1*	AROmatic amino acid requiring	0.83613	0.83965
YGL148W	*ARO2*	AROmatic amino acid requiring	0.83613	0.83965
YPR060C	*ARO7*	AROmatic amino acid requiring	0.83527	0.83859
YNL220W	*ADE12*	ADEnine requiring	0.82882	0.79545
YJR073C	*OPI3*	OverProducer of Inositol	0.81508	0.86237
…	…	…	…	

Supplementary Table 3. The 49 NED genes present in all the *MMN*s in Aerobic Conditions (3 media) and the corresponding 7 Mandatory Genes in Anaerobic Conditions (3 media). For each gene the frequency in the other conditions is reported (considering the 1% Growth rate threshold results). All the mandatory genes for anaerobic MNs are also mandatory for the aerobic MNs.

NED **Genes in Aerobic Conditions**	**Standard Names**	**Name Description**	**Frequency (Anaerobic)**
Q0045	*COX1*	Cytochrome c OXidase	0
Q0080	*ATP8*	ATP synthase	0.005126015
Q0085	*ATP6*	ATP synthase	0.005126015
Q0105	*COB*	CytochrOme B	0
Q0130	*OLI1*	OLIgomycin resistance	0.005126015
Q0250	*COX2*	Cytochrome c OXidase	0
Q0275	*COX3*	Cytochrome c OXidase	0
YAL044C	*GCV3*	GlyCine cleaVage	0.179837676
YBL045C	*COR1*	CORe protein of QH2 cytochrome c reductase	0
YBL099W	*ATP1*	ATP synthase	0.005126015
YBR039W	*ATP3*	ATP synthase	0.005126015
YBR126C	*TPS1*	Trehalose-6-Phosphate Synthase	1
YBR263W	*SHM1*	Serine HydroxyMethyltransferase	0.327637762
YDL067C	*COX9*	Cytochrome c OXidase	0
YDR019C	*GCV1*	GlyCine cleaVage	0.179837676
YDR074W	*TPS2*	Trehalose-6-Phosphate Synthase/phosphatase	1
YDR127W	*ARO1*	AROmatic amino acid requiring	0.67407091
YDR298C	*ATP5*	ATP synthase	0.005126015
YDR377W	*ATP17*	ATP synthase	0.005126015
YDR529C	*QCR7*	ubiQuinol-cytochrome C oxidoReductase	0
YEL024W	*RIP1*	Rieske Iron-sulfur Protein	0
YER014W	*HEM14*	HEMe biosynthesis	0
YER026C	*CHO1*	CHOline requiring	1
YFL018C	*LPD1*	LiPoamide Dehydrogenase	0.27765912
YFR033C	*QCR6*	ubiQuinol-cytochrome C oxidoReductase	0
YGL148W	*ARO2*	AROmatic amino acid requiring	0.67407091
YGR183C	*QCR9*	ubiQuinol-cytochrome C oxidoReductase	0
YGR204W	*ADE3*	ADEnine requiring	1
YHR001W-A	*QCR10*	ubiQuinol-cytochrome C oxidoReductase	0
YJL166W	*QCR8*	ubiQuinol-cytochrome C oxidoReductase	0
YJR121W	*ATP2*	ATP synthase	0.005126015
YKL016C	*ATP7*	ATP synthase	0.005126015
YKL067W	*YNK1*	Yeast Nucleoside diphosphate Kinase	1
YKR067W	*GPT2*	Glycerol-3-Phosphate acylTransferase	1
YLR058C	*SHM2*	Serine HydroxyMethyltransferase	0.749252456
YLR295C	*ATP14*	ATP synthase	0.005126015
YML081C-A	*ATP18*	ATP synthase	0.005126015
YMR189W	*GCV2*	GlyCine cleaVage	0.179837676
YMR205C	*PFK2*	PhosphoFructoKinase	1
YMR267W	*PPA2*	PyroPhosphatAse	0.042716788
YOR065W	*CYT1*	CYTochrome c1	0
YPL078C	*ATP4*	ATP synthase	0.005126015
YPL172C	*COX10*	Cytochrome c OXidase	0
YPL271W	*ATP15*	ATP synthase	0.005126015
YPR060C	*ARO7*	AROmatic amino acid requiring	0.672362238
YPR191W	*QCR2*	QH2:cytochrome-C oxidoReductase	0
YKL029C	*MAE1*	MAlic Enzyme	0.593336181
YPR160W	*GPH1*	Glycogen PHosphorylase	0.591200342
YPL262W	*FUM1*	FUMarase	0.548056386
**Magnificent 7 Genes in Anaerobic Conditions**	**Standard Names**	**Name Description**	**Frequency (Aerobic)**
YBR126C	*TPS1*	Trehalose-6-Phosphate Synthase	1
YDR074W	*TPS2*	Trehalose-6-Phosphate Synthase/phosphatase	1
YER026C	*CHO1*	CHOline requiring	1
YGR204W	*ADE3*	ADEnine requiring	1
YKL067W	*YNK1*	Yeast Nucleoside diphosphate Kinase	1
YKR067W	*GPT2*	Glycerol-3-Phosphate acylTransferase	1
YMR205C	*PFK2*	PhosphoFructoKinase	1

Supplementary Table 4. The Genes with the greatest frequency difference between Aerobic and Anaerobic external conditions (considering the 1% Growth rate threshold results only).

Systematic Names	Standard Gene Names	Name Description	Aerobic	Anaerobic	Difference
Q0045	*COX1*	Cytochrome c OXidase	1	0	1
Q0105	*COB*	CytochrOme B	1	0	1
Q0250	*COX2*	Cytochrome c OXidase	1	0	1
Q0275	*COX3*	Cytochrome c OXidase	1	0	1
YBL045C	*COR1*	CORe protein of QH2 cytochrome c reductase	1	0	1
YDL067C	*COX9*	Cytochrome c OXidase	1	0	1
YDR529C	*QCR7*	ubiQuinol-cytochrome C oxidoReductase	1	0	1
YEL024W	*RIP1*	Rieske Iron-sulfur Protein	1	0	1
YER014W	*HEM14*	HEMe biosynthesis	1	0	1
YFR033C	*QCR6*	ubiQuinol-cytochrome C oxidoReductase	1	0	1
YGR183C	*QCR9*	ubiQuinol-cytochrome C oxidoReductase	1	0	1
YHR001W-A	*QCR10*	ubiQuinol-cytochrome C oxidoReductase	1	0	1
YJL166W	*QCR8*	ubiQuinol-cytochrome C oxidoReductase	1	0	1
YOR065W	*CYT1*	CYTochrome c1	1	0	1
YPL172C	*COX10*	Cytochrome c OXidase	1	0	1
YPR191W	*QCR2*	QH2:cytochrome-C oxidoReductase	1	0	1
Q0080	*ATP8*	ATP synthase	1	0.005126015	0.994873985
Q0085	*ATP6*	ATP synthase	1	0.005126015	0.994873985
Q0130	*OLI1*	OLIgomycin resistance	1	0.005126015	0.994873985
YBL099W	*ATP1*	ATP synthase	1	0.005126015	0.994873985
YBR039W	*ATP3*	ATP synthase	1	0.005126015	0.994873985
YDR298C	*ATP5*	ATP synthase	1	0.005126015	0.994873985
YDR377W	*ATP17*	ATP synthase	1	0.005126015	0.994873985
YJR121W	*ATP2*	ATP synthase	1	0.005126015	0.994873985
YKL016C	*ATP7*	ATP synthase	1	0.005126015	0.994873985
YLR295C	*ATP14*	ATP synthase	1	0.005126015	0.994873985
YML081C-A	*ATP18*	ATP synthase	1	0.005126015	0.994873985
YPL078C	*ATP4*	ATP synthase	1	0.005126015	0.994873985
YPL271W	*ATP15*	ATP synthase	1	0.005126015	0.994873985
YMR267W	*PPA2*	PyroPhosphatAse	1	0.042716788	0.957283212
YAL044C	*GCV3*	GlyCine cleaVage	1	0.179837676	0.820162324
YDR019C	*GCV1*	GlyCine cleaVage	1	0.179837676	0.820162324
YMR189W	*GCV2*	GlyCine cleaVage	1	0.179837676	0.820162324
YFL018C	*LPD1*	LiPoamide Dehydrogenase	1	0.27765912	0.72234088
YOL126C	*MDH2*	Malate DeHydrogenase	0.701511879	0.002563007	0.698948872
YDR148C	*KGD2*	alpha-KetoGlutarate Dehydrogenase	0.694168467	0.009397693	0.684770773
YIL125W	*KGD1*	alpha-KetoGlutarate Dehydrogenase	0.694168467	0.009397693	0.684770773
YNL037C	*IDH1*	Isocitrate DeHydrogenase	0.717926566	0.037590773	0.680335793
YOR136W	*IDH2*	Isocitrate DeHydrogenase	0.717926566	0.037590773	0.680335793
YBR263W	*SHM1*	Serine HydroxyMethyltransferase	1	0.327637762	0.672362238
YNL280C	*ERG24*	ERGosterol biosynthesis	0.666522678	0	0.666522678

YOR388C	*FDH1*	Formate DeHydrogenase	0.666522678	0.001708672	0.664814007
YHR037W	*PUT2*	Proline UTilization	0.688552916	0.055531824	0.633021092
YLR142W	*PUT1*	Proline UTilization	0.688552916	0.055531824	0.633021092

Supplementary Table 5. The Genes with the greatest frequency difference between Minimal and YPD Aerobic and Anaerobic external conditions (considering the 1% Growth rate threshold results only).

Gene			YPD	Min	AnYPD	AnMin
YNL280C	*ERG24*	ERGosterol biosynthesis	0	1	0	0
YOR388C	*FDH1*	Formate DeHydrogenase	0	1	0.0025031	0
YFL030W	*AGX1*	Alanine:Glyoxylate aminotrans(X)ferase	1	0	0.011264	0
YML035C	*AMD1*	AMP Deaminase	0.027202	0.063492	0.043805	0.97919
YDL238C	*GUD1*	GUanine Deaminase	0.96244	0	0.075094	0
YJR148W	*BAT2*	Branched-chain Amino acid Transaminase	0.9456	1	0.090113	1
YDL080C	*THI3*	THIamine metabolism	0.8614	0	0.045056	0
YHR002W	*LEU5*	LEUcine biosynthesis	0	0.80688	0	0
YER065C	*ICL1*	IsoCitrate Lyase	1	0.010582	0.18523	0
YHR037W	*PUT2*	Proline UTilization	1	0.046296	0.15144	0
YLR142W	*PUT1*	Proline UTilization	1	0.046296	0.15144	0
YHR018C	*ARG4*	ARGinine requiring	0.75	1	0.021277	1
YOL058W	*ARG1*	ARGinine requiring	0.75	1	0.021277	1
YEL047C	*FRD1*	Fumarate ReDuctase	1	0.21958	0.20651	0.11964
YDL215C	*GDH2*	Glutamate DeHydrogenase	0.82642	0.063492	0.070088	0
YCR053W	*THR4*	THReonine requiring	0.61658	1	0	1
YHR025W	*THR1*	THReonine requiring	0.61658	1	0	1
YDR158W	*HOM2*	HOMoserine requiring	0.62435	1	0.008761	1
YER052C	*HOM3*	HOMoserine requiring	0.62435	1	0.008761	1
YJR139C	*HOM6*	HOMoserine requiring	0.62435	1	0.008761	1
YKL120W	*OAC1*	OxaloAcetate Carrier	0.007772	0.16138	0.010013	0.76593
YFL018C	*LPD1*	LiPoamide Dehydrogenase	1	1	0.098874	0.69571
YDR516C	*EMI2*	Early Meiotic Induction	1	0.77513	0.20526	1
YBR221C	*PDB1*	Pyruvate Dehydrogenase Beta subunit	0.018135	1	0.0025031	0.42003
YER178W	*PDA1*	Pyruvate Dehydrogenase Alpha	0.018135	1	0.0025031	0.42003
YGR193C	*PDX1*	Pyruvate Dehydrogenase complex protein X	0.018135	1	0.0025031	0.42003
YNL071W	*LAT1*	""	0.018135	1	0.0025031	0.42003
YML042W	*CAT2*	Carnitine AcetylTransferase	0	0	0.020025	0.57997
YPR160W	*GPH1*	Glycogen PHosphorylase	0.8899	1	0.34418	1
YJR095W	*SFC1*	Succinate-Fumarate Carrier	0.82254	0.52381	0.042553	0.28218

Supplementary Table 6. Quantitative analysis of the genes in the *MMN*s using Functional Category and Compartment annotations. In the first two columns are the number of genes that can be attributed to the specific category and the percentage in the WT genome. The next two columns are relative to the SD Media mean values in the MNs, with the average percentage of genes that were turned off. In the next column the difference in the KO values is reported (1% Growth rate threshold). The next three columns are analogous for the minimal media.

**Functional categories**	**# Genes in the WT**	**Relative Frequency**	**Mean of KO Perc. (SD)**	**Avg KO Perc. (SD An)**	**Diff. in KO**	**Avg KO Perc.(Min)**	**Avg KO Perc. (Min. An.)**	**Diff in KO**
transferase activity	320	28.24	64.55	70.44	-5.89	60.44	61.43	-0.98
oxidoreductase activity	210	18.53	68.29	84.15	-15.86	64.91	73.35	-8.44
hydrolase activity	192	16.95	86.46	89.09	-2.62	85.52	85.98	-0.46
transmembrane transport	146	12.89	86.77	88.97	-2.19	90.00	89.15	0.85
lipid metabolic process	116	10.24	66.71	68.07	-1.36	65.85	66.66	-0.81
ligase activity	83	7.33	55.27	60.17	-4.90	49.64	48.23	1.41
lyase activity	83	7.33	67.27	73.96	-6.69	58.26	57.72	0.54
kinase activity	76	6.71	64.23	69.96	-5.73	61.81	61.30	0.51
transmembrane transporter activity	69	6.09	85.91	86.02	-0.11	92.38	91.63	0.75
ion transport	66	5.83	69.57	92.69	-23.12	69.41	92.02	-22.61
transferase activity, transferring glycosyl groups	64	5.65	61.75	62.61	-0.86	59.27	59.24	0.02
carbohydrate metabolic process	59	5.21	84.19	84.53	-0.34	80.10	78.58	1.52
unassigned	48	4.24	59.32	87.84	-28.51	58.95	81.80	-22.85
biological_process	38	3.35	90.73	93.53	-2.80	92.45	92.59	-0.14
protein glycosylation	36	3.18	67.97	68.33	-0.36	68.12	68.03	0.09
amino acid transport	34	3.00	89.94	90.98	-1.04	94.19	92.45	1.74
isomerase activity	29	2.56	51.53	56.32	-4.79	45.50	44.83	0.67
tRNA aminoacylation for protein translation	29	2.56	43.18	43.04	0.14	43.10	43.19	-0.08
molecular_function	28	2.47	85.56	93.25	-7.69	89.06	92.00	-2.94
hydrolase activity, acting on glycosyl bonds	27	2.38	100.00	98.60	1.40	100.00	100.00	0.00
carbohydrate transport	26	2.29	92.31	92.28	0.02	92.29	92.31	-0.02
mRNA binding	25	2.21	67.54	65.59	1.95	57.90	58.67	-0.77
ATPase activity	23	2.03	56.47	86.66	-30.19	56.51	84.79	-28.28
cellular amino acid metabolic process	23	2.03	69.17	92.10	-22.94	59.32	57.95	1.37
RNA binding	21	1.85	60.30	69.36	-9.06	65.02	65.90	-0.88
methyltransferase activity	20	1.77	89.00	88.41	0.59	85.00	85.00	0.00
nucleotidyltransferase activity	18	1.59	51.09	55.54	-4.45	50.00	50.00	0.00
phosphatase activity	11	0.97	100.00	100.00	0.00	100.00	100.00	0.00
Others	144	12.71	85.15	88.03	-2.88	82.55	85.07	-2.52
**Compartments**								
cytoplasm	470	41.48	67.02	73.11	-6.09	60.58	60.17	0.41
membrane	449	39.63	70.67	80.92	-10.25	71.99	80.25	-8.26
mitochondrion	376	33.19	63.70	82.33	-18.63	61.78	73.97	-12.19
nucleus	198	17.48	69.74	74.30	-4.56	64.75	64.50	0.25
endoplasmic reticulum	181	15.98	64.39	66.59	-2.20	65.68	67.16	-1.48
plasma membrane	153	13.50	78.03	78.27	-0.24	80.21	79.32	0.89

unassigned	86	7.59	82.56	85.85	-3.30	80.60	80.27	0.33
vacuole	61	5.38	91.76	91.91	-0.15	94.20	93.62	0.58
Golgi apparatus	40	3.53	80.84	82.62	-1.78	80.32	80.89	-0.57
peroxisome	32	2.82	94.15	97.94	-3.79	96.72	93.83	2.89
extracellular region	21	1.85	93.93	97.03	-3.11	95.17	95.11	0.05
ribosome	9	0.79	57.63	69.90	-12.27	67.00	67.92	-0.92
cytoskeleton	3	0.26	67.02	73.11	-6.09	100.00	100.00	0.00

Supplementary Figure 1. For each condition, the *MMN*s active genes number using a Growth rate threshold of 1% or 10% is reported. On average less genes are required for the less strict bound. The minimal networks in minimal media are significantly larger even with the looser threshold. Notably, in SD medium the *MMN*s have more diverse sizes. Differences between the distributions in SD and YPD anaerobic media are less distinct.

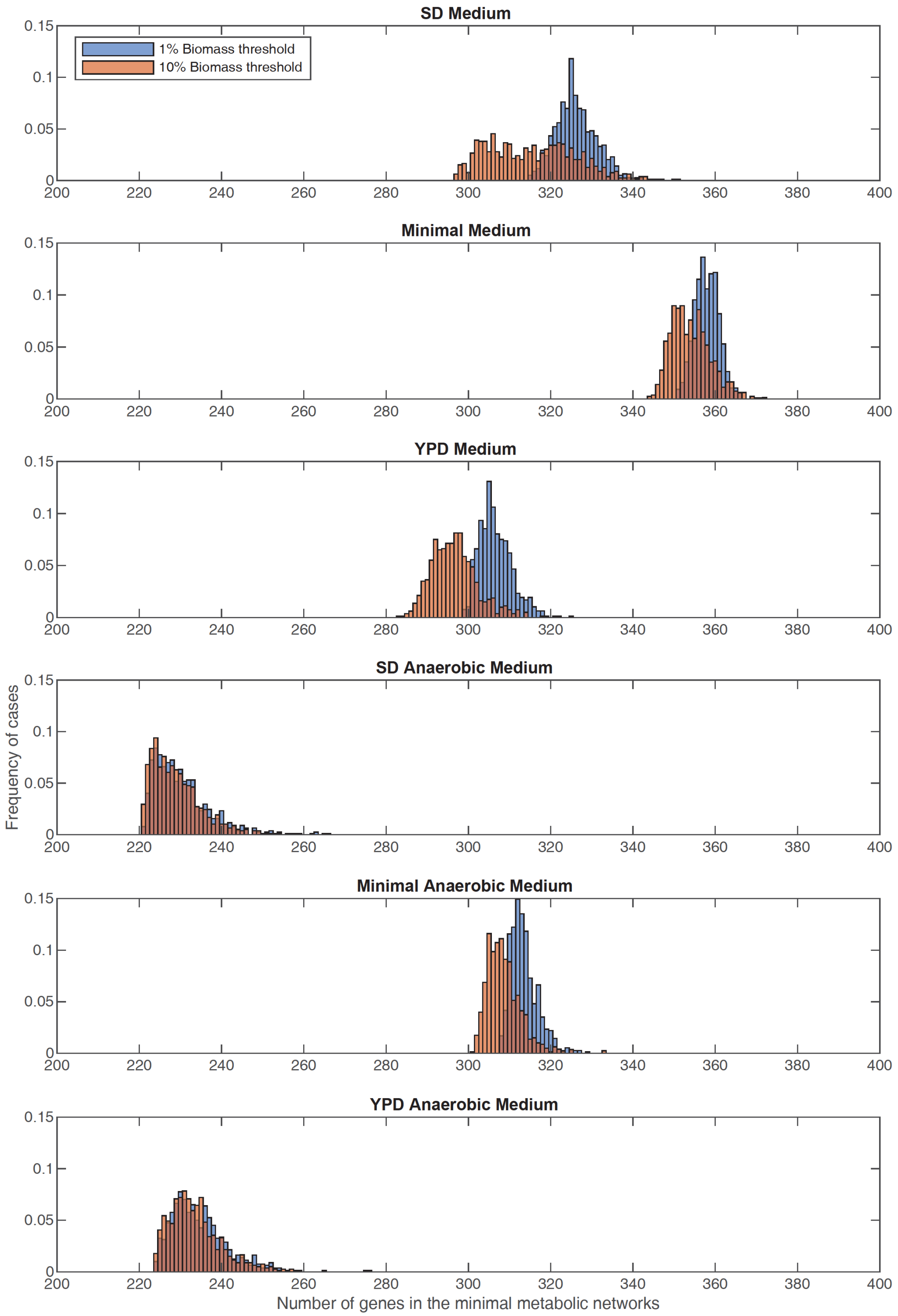

Supplementary Figure 2. We considered the genes annotated as transporter-related to highlight that, in SD medium, the number of such genes excluded from the *MMN*s is lower than in other conditions. Conversely, the number of KOs in this category is higher in SD *anaerobic* medium than that under anaerobiosis. (Supplementary Data 5 gives a full list of transporter genes and their biological functions.)

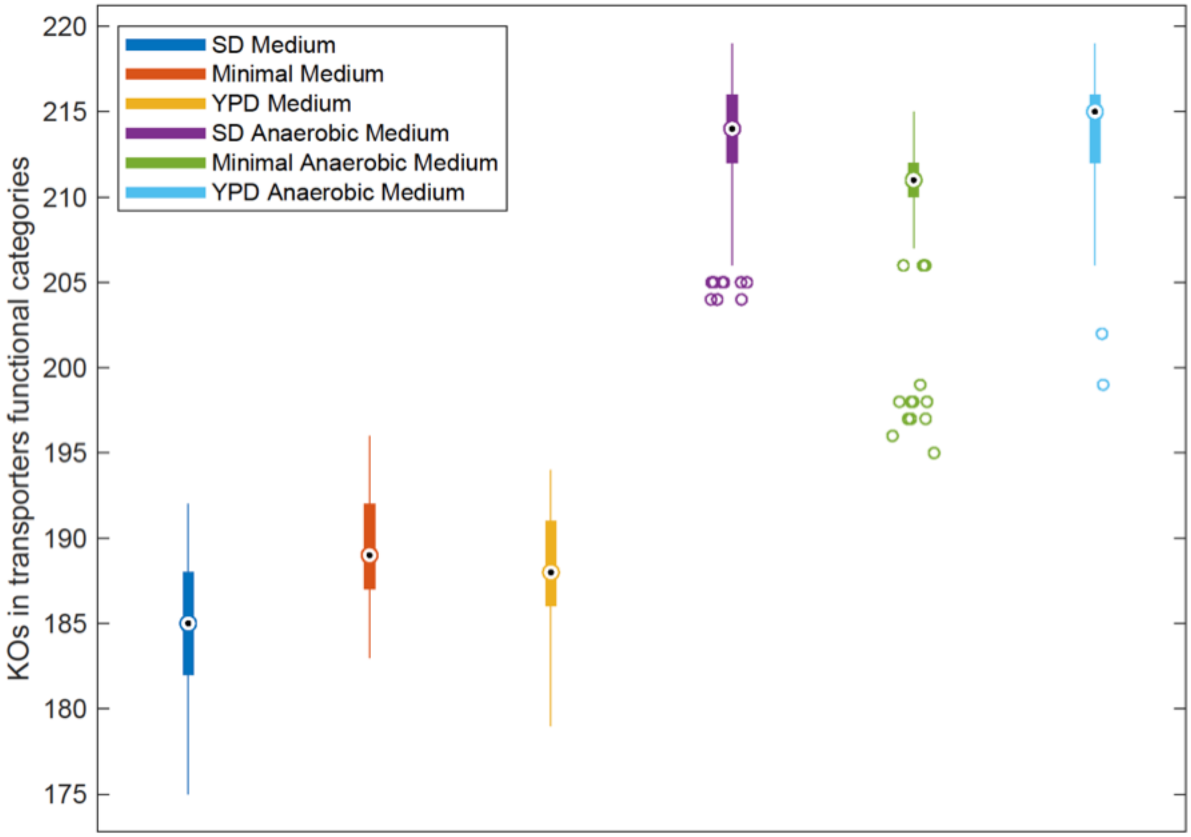

Supplementary Figure 3. Frequency of knockout genes in the minimal metabolic networks divided by functional categories and compartments.

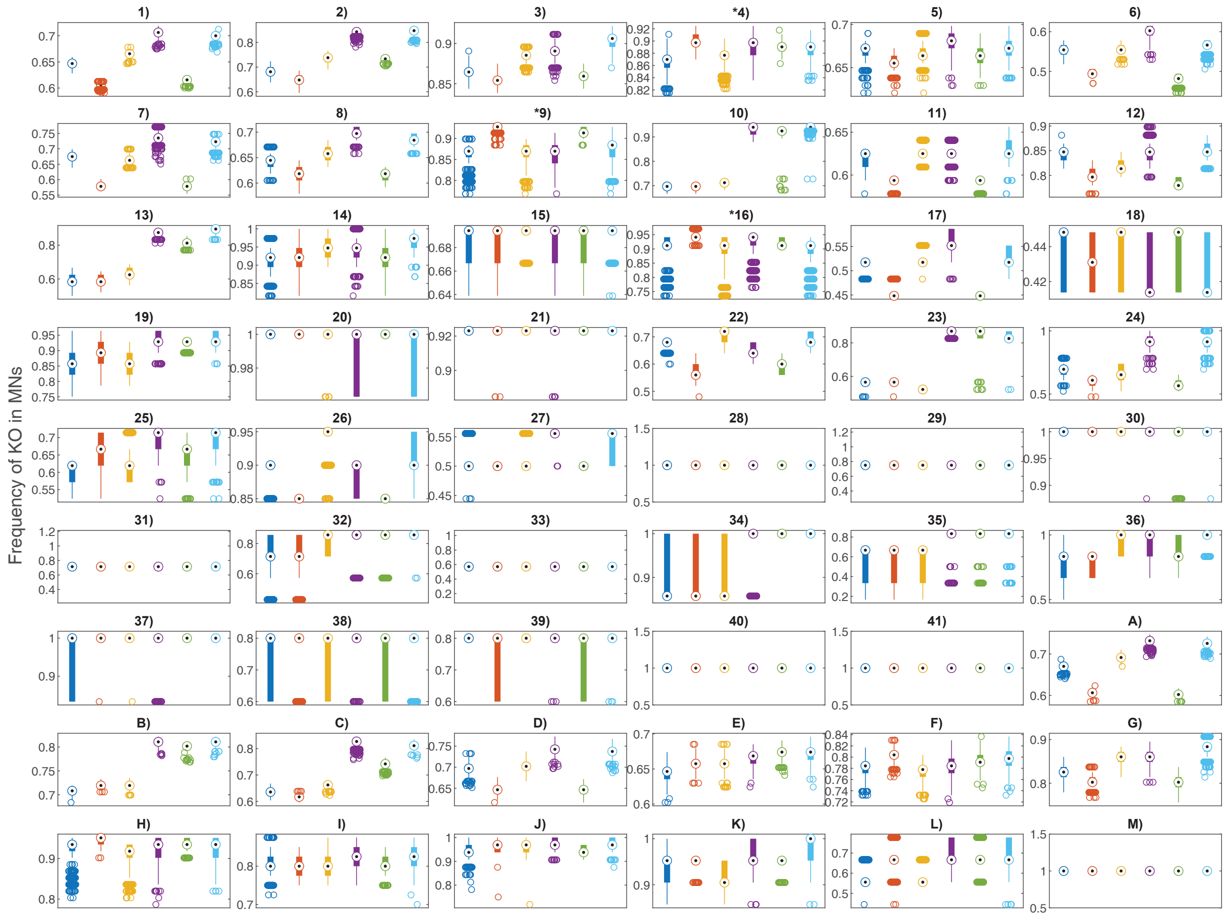

1) transferase activity
2) oxidoreductase activity
3) hydrolase activity
4) ***transmembrane transport**
5) lipid metabolic process
6) ligase activity
7) lyase activity
8) kinase activity
9) ***transmembrane transporter activity**
10) ion transport
11) transferase activity, transferring glycosyl groups
12) carbohydrate metabolic process
13) unassigned
14) biological_process
15) protein glycosylation
16) ***amino acid transport**
17) isomerase activity
18) tRNA aminoacylation for protein translation
19) molecular_function
20) hydrolase activity, acting on glycosyl bonds
21) carbohydrate transport
22) mRNA binding
23) ATPase activity
24) cellular amino acid metabolic process
25) RNA binding
26) methyltransferase activity
27) nucleotidyltransferase activity
28) phosphatase activity
29) mitochondrial translation
30) peptidase activity
31) RNA modification
32) enzyme regulator activity
33) tRNA processing
34) unfolded protein binding
35) cellular respiration
36) pseudohyphal growth
37) response to oxidative stress
38) DNA binding
39) DNA repair
40) chromatin organization
41) endocytosis

A) cytoplasm
B) membrane
C) mitochondrion
D) nucleus
E) endoplasmic reticulum
F) plasma membrane
G) unassigned
H) vacuole
I) Golgi apparatus
J) peroxisome
K) extracellular region
L) ribosome
M) cytoskeleton

SD Medium
Minimal Medium
YPD Medium
SD Anaerobic Medium
Minimal Anaerobic Medium
YPD Anaerobic Medium

Supplementary Figure 4. Growth characteristics of double deletant strains (strains with YPD medium in the top plots, strains with SD medium in the bottom plots). Data are presented as mean values +/- SD (standard deviation), with 3 technical replicates and 2 biological replicates (except for *suc2Δ/chs1Δ*, for which only one transformant was recovered). Source data is provided as a single Source Data file: Fig 5 and SI Figs 4-6 Source data for experimental parts.xlsx.

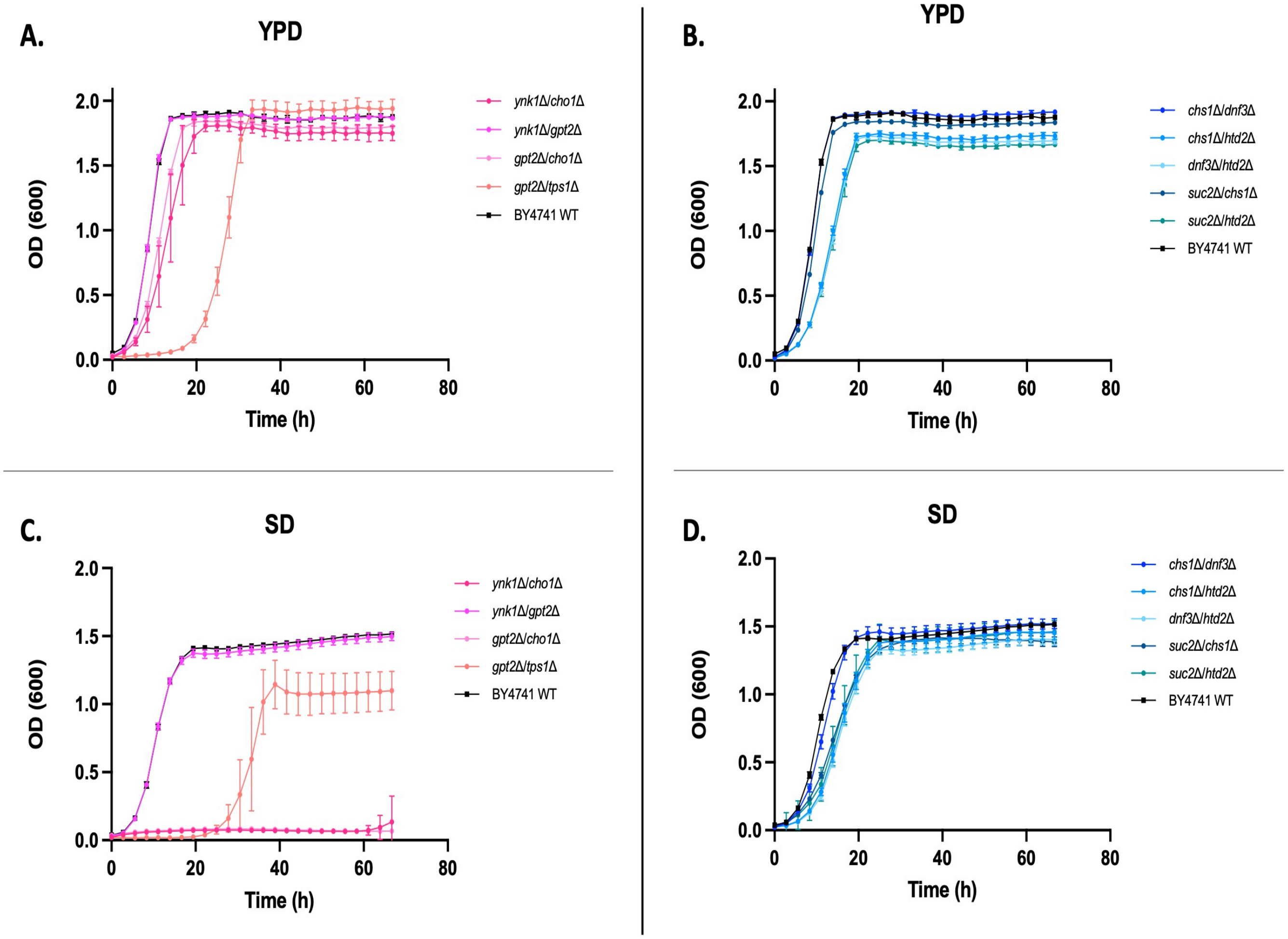

Supplementary Figure 5. Growth characteristics of triple deletant strains (strains with YPD medium in the top plot, strains with SD medium in the bottom plot). Data are presented as mean values +/- SD (standard deviation), with 3 technical replicates and 2 biological replicates (except for *chs1Δ/dnf3Δ/suc2Δ,* for which only one transformant was recovered). Source data is provided as a single Source Data file: Fig 5 and SI Figs 4-6 Source data for experimental parts.xlsx.

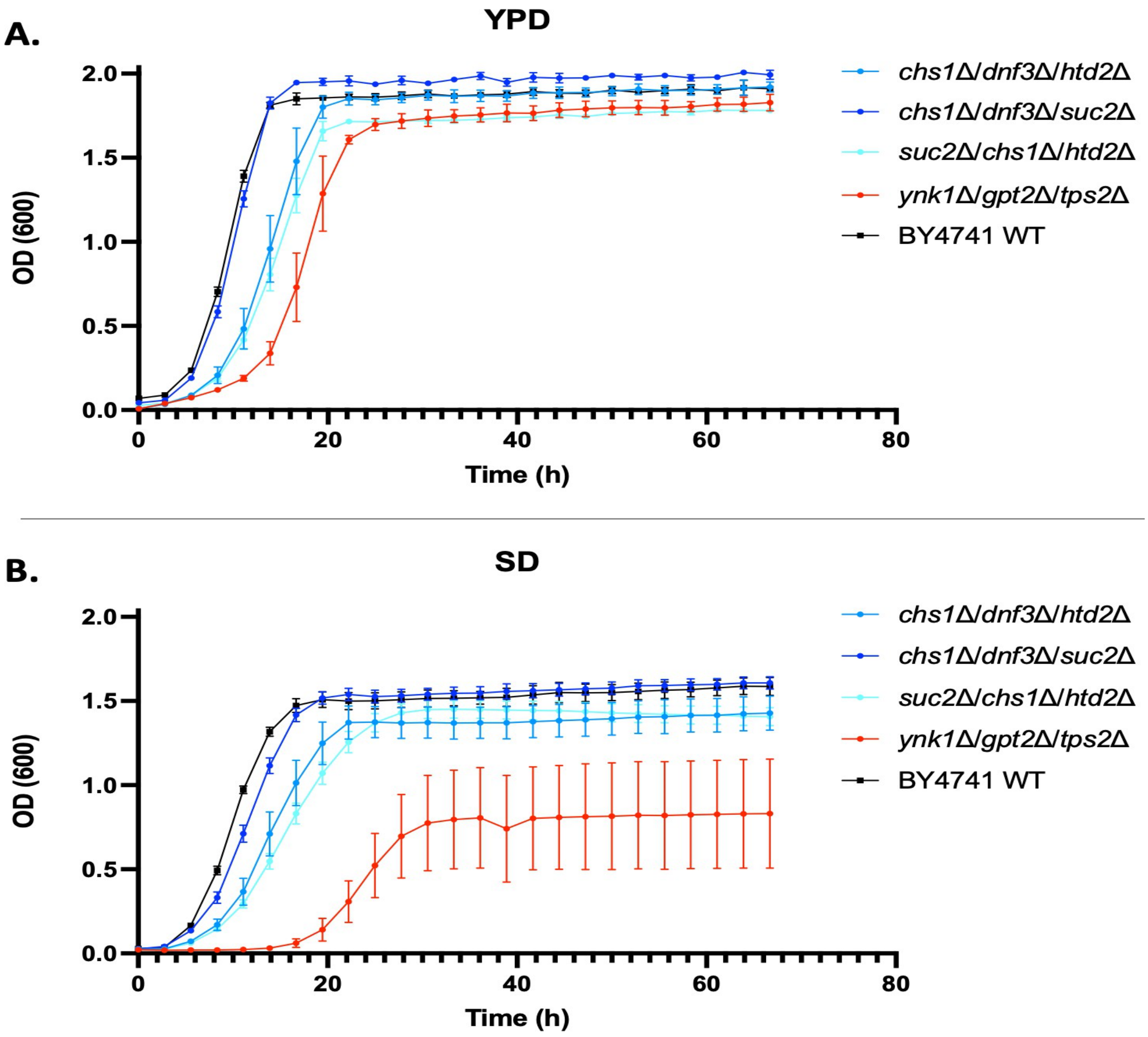

Supplementary Figure 6. Growth characteristics of quadruple deletant strains (strains with YPD medium in the top plot, strains with SD medium in the bottom plot). Data are presented as mean values +/- SD (standard deviation), with 3 technical replicates and 2 biological replicates (except for *chs1Δ/dnf3Δ/htd2Δ/zta1Δ* and *chs1Δ/dnf3Δ/suc2Δ/zta1Δ*, for which only one transformant was recovered respectively). Source data is provided as a single Source Data file: Fig 5 and SI Figs 4-6 Source data for experimental parts.xlsx.

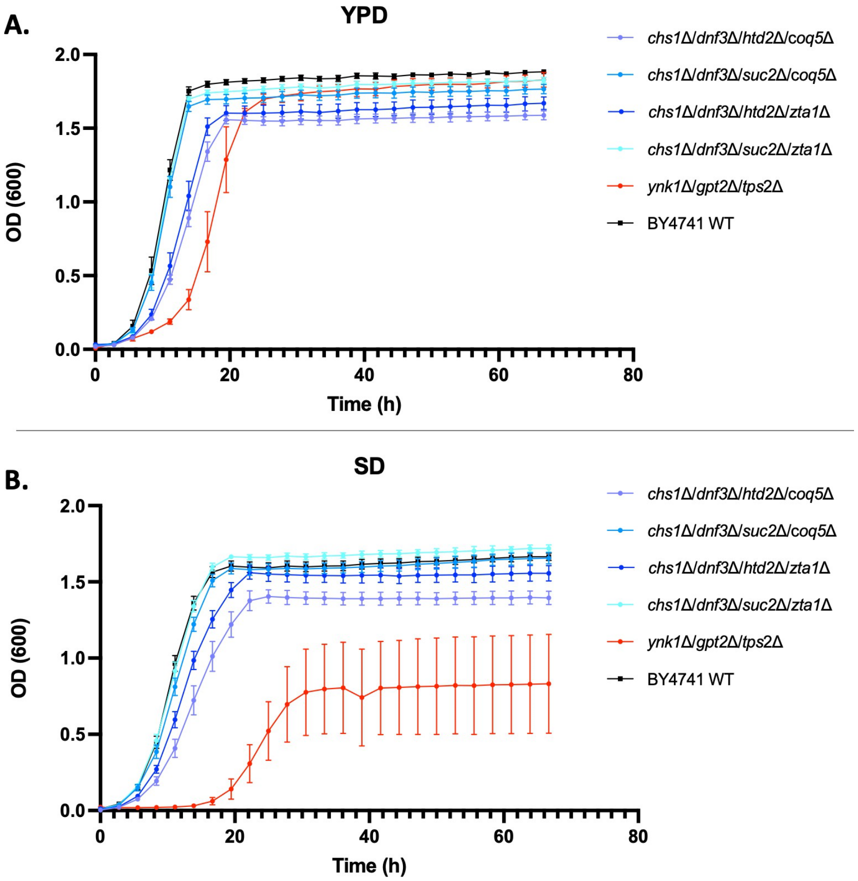

Supplementary Figure 7. Uncropped images of the growth characteristics of deletant strains (YPD medium in the left plot, SD medium in the right plot). The experiment was performed twice, yielding the same results each time.

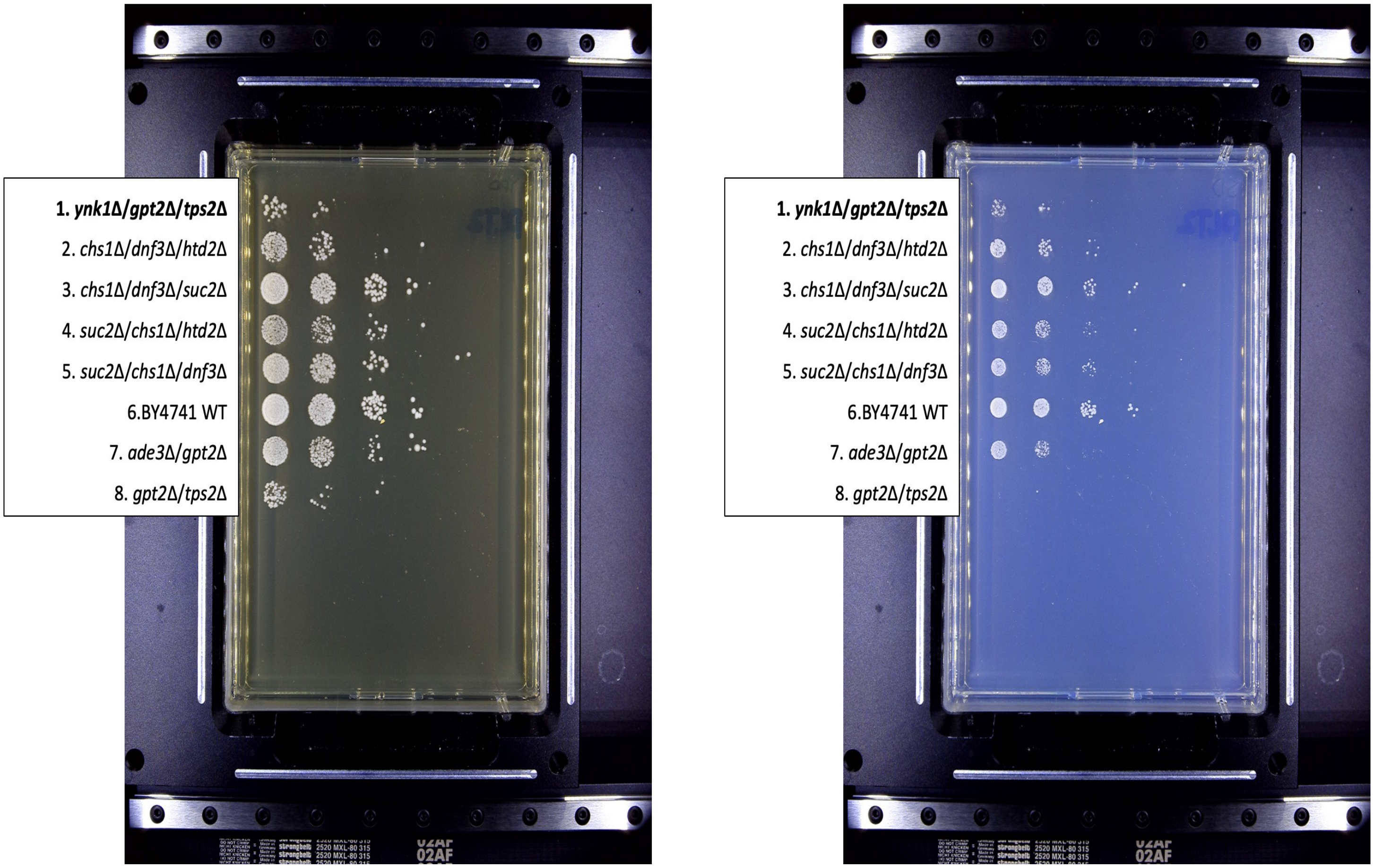

Supplementary Figure 8. *S. cerevisiae* metabolic network of the computational model used (Yeast 8.3.1).
The JSON files used to generate Figs. 8-12, and which readers can use to generate their own metabolic maps may be downloaded from:
https://github.com/GiuseppeNicosia1/MinimalNetwork_CompleteCode/blob/main/escher-metabolic-maps.zip

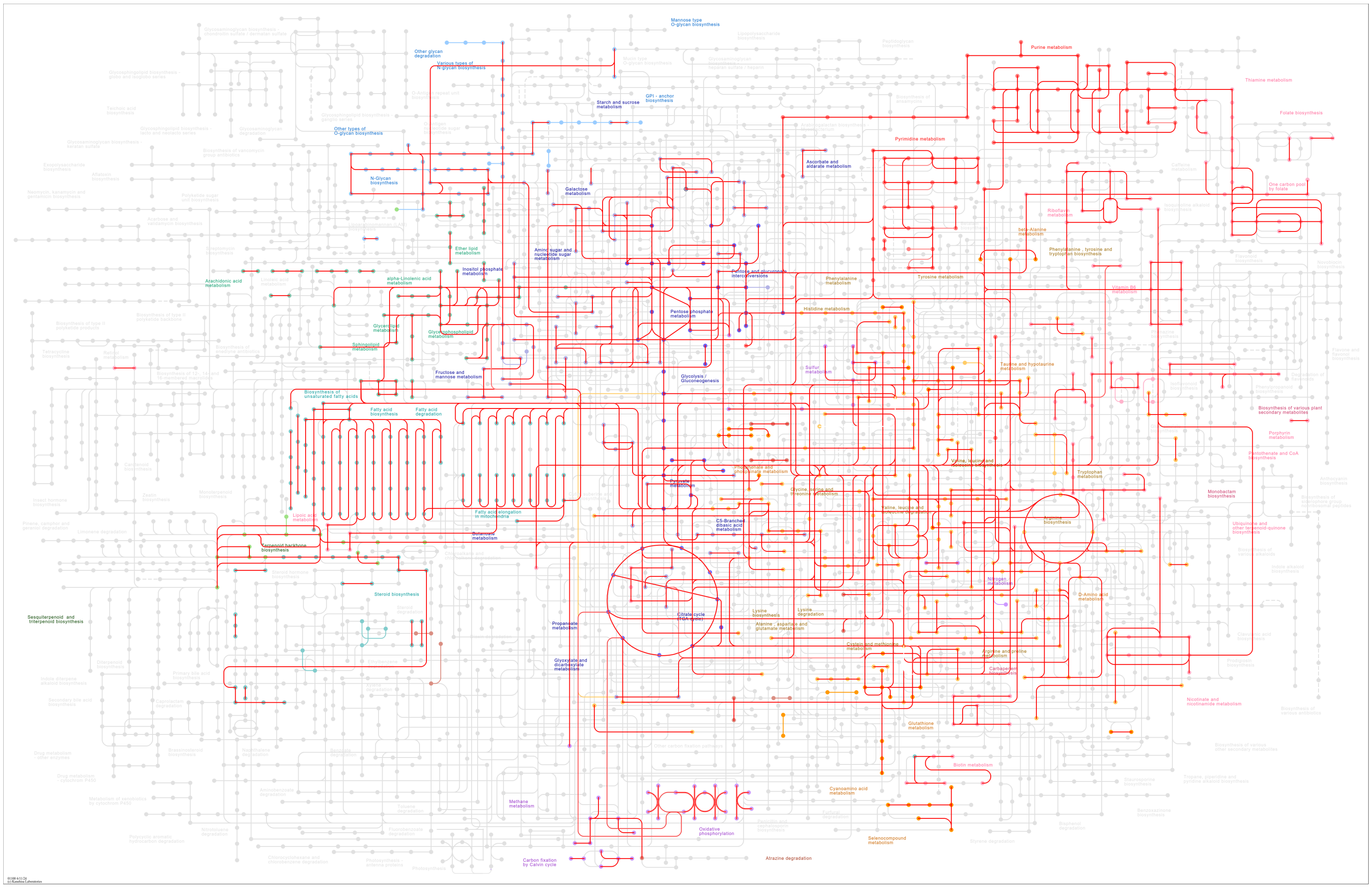

Supplementary Figure 9. The metabolic network of *S. cerevisiae* as present in the KEGG database (https://www.genome.jp/kegg/).

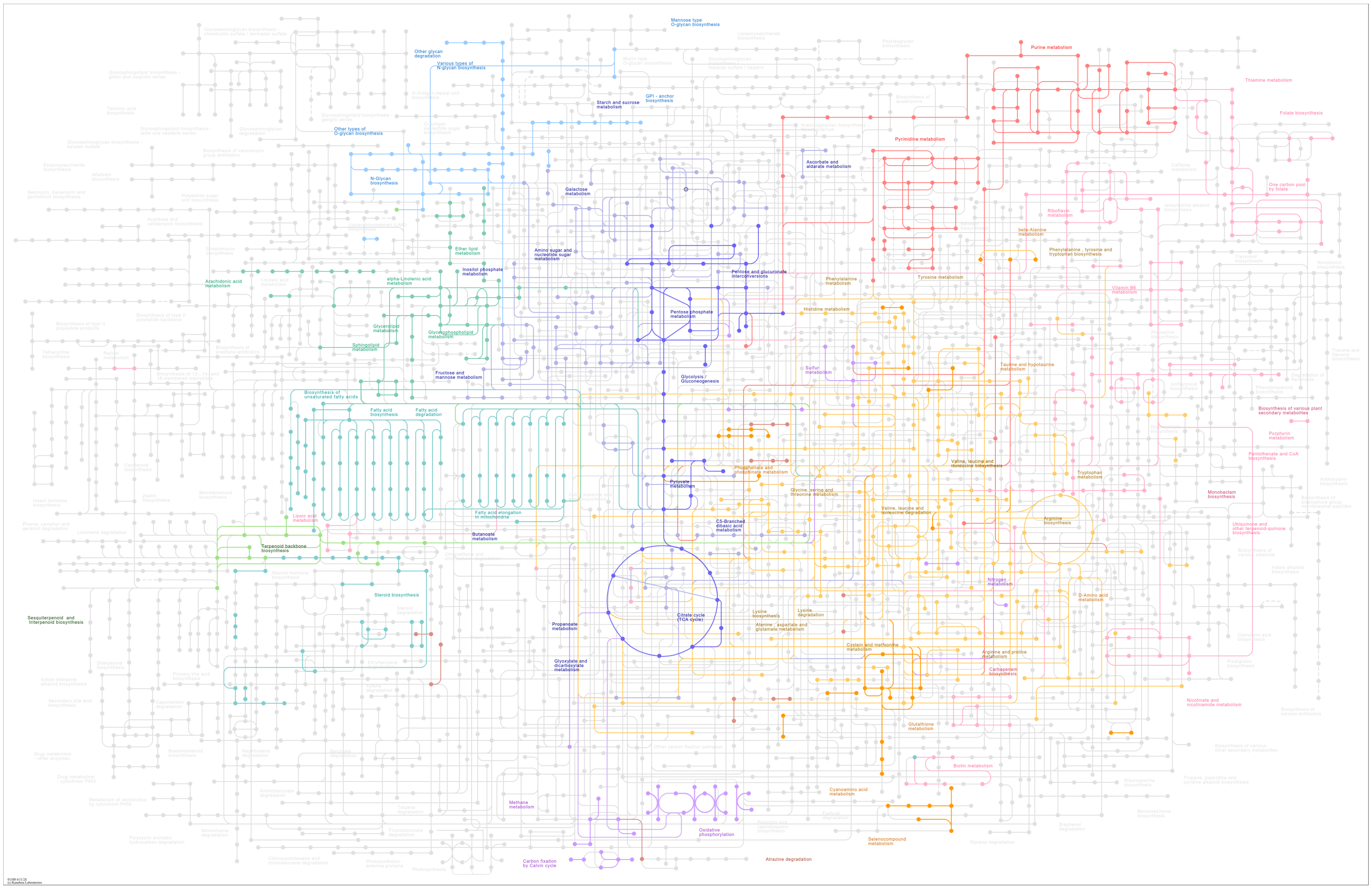

Supplementary Figure 10. This figure shows the flux change (obtained using Escher) of a single minimal metabolic network using the YPD medium. As can be appreciated from the image, some fluxes show well-marked colours (such as red), this indicates a significant difference in the flux in question between the minimal metabolic network obtained and the wild type. The fluxes coloured in red represent a higher difference of value than the fluxes coloured in blue.

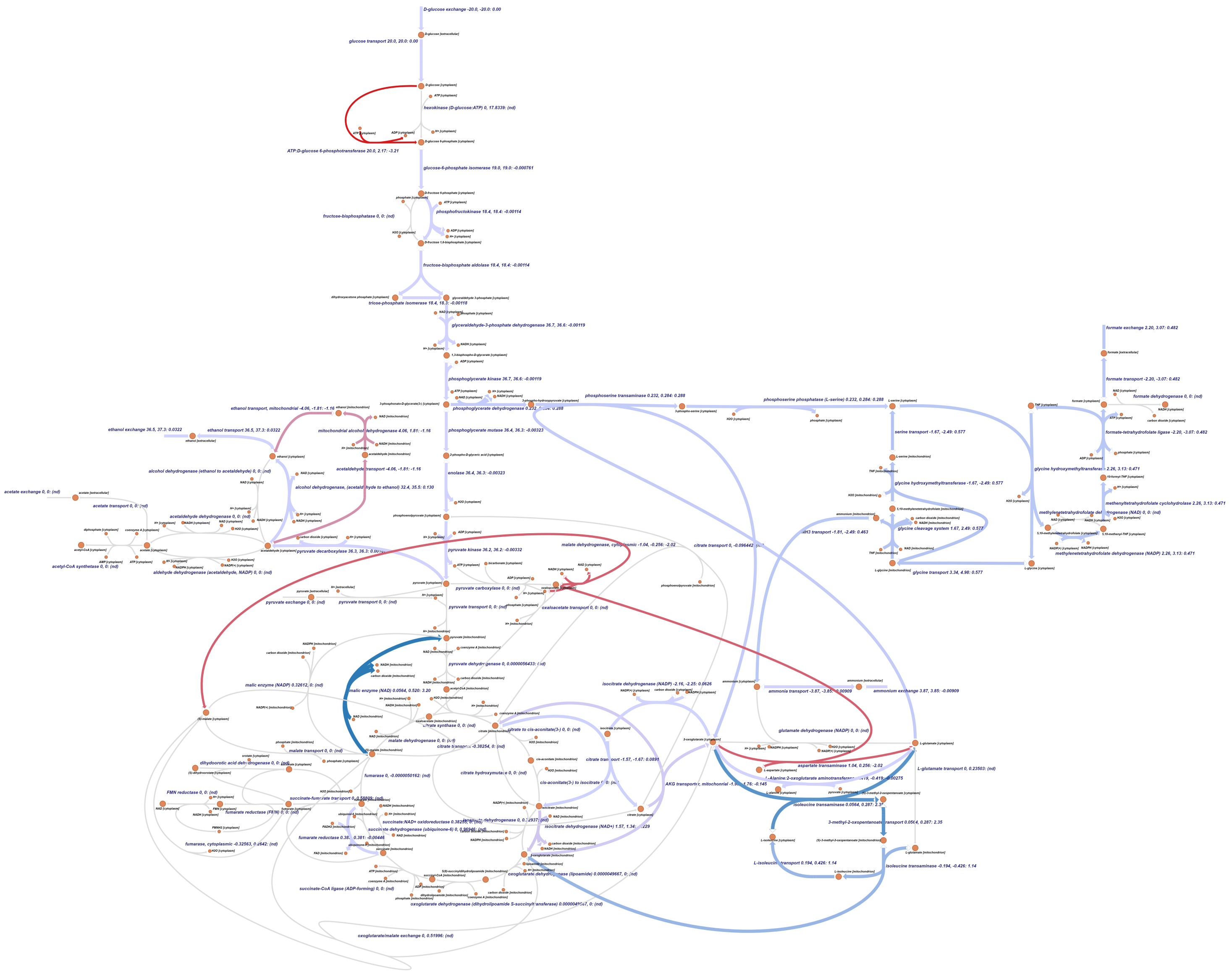

Supplementary Figure 11. Metabolic map obtained from KEGG (https://www.genome.jp/kegg/). Some pathways linked to metabolic genes are highlighted: in black the essential genes in the model, in blue the Network Efficiency Determinants (*NED*s) in aerobic conditions, in red the NEDs present in all media.

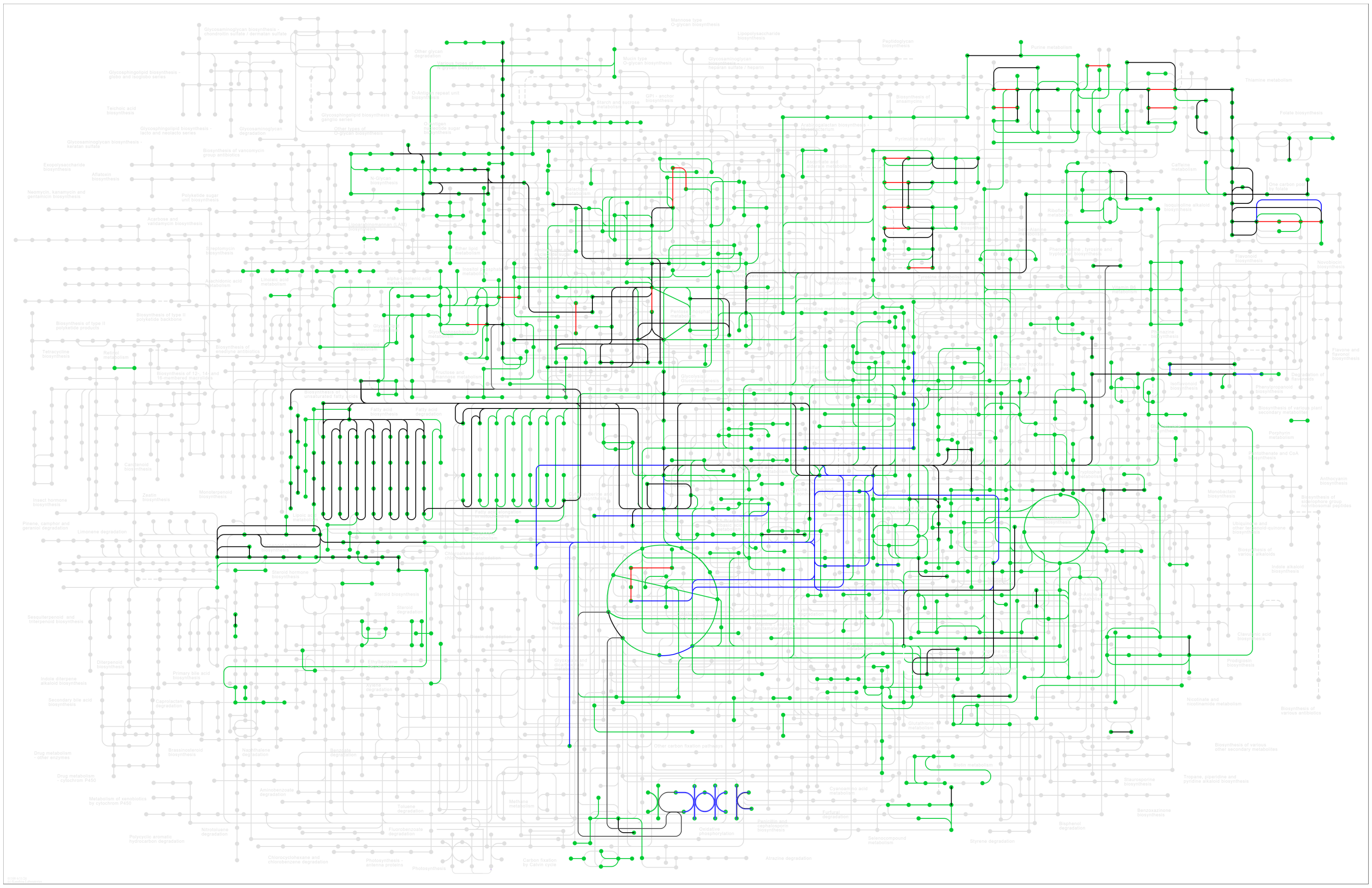

Supplementary Figure 12. Metabolic map obtained from KEGG (https://www.genome.jp/kegg/). Some pathways linked to metabolic genes are highlighted: in black the essential genes in the model, in blue the Network Efficiency Determinants (*NEDs*) in the YPD medium.

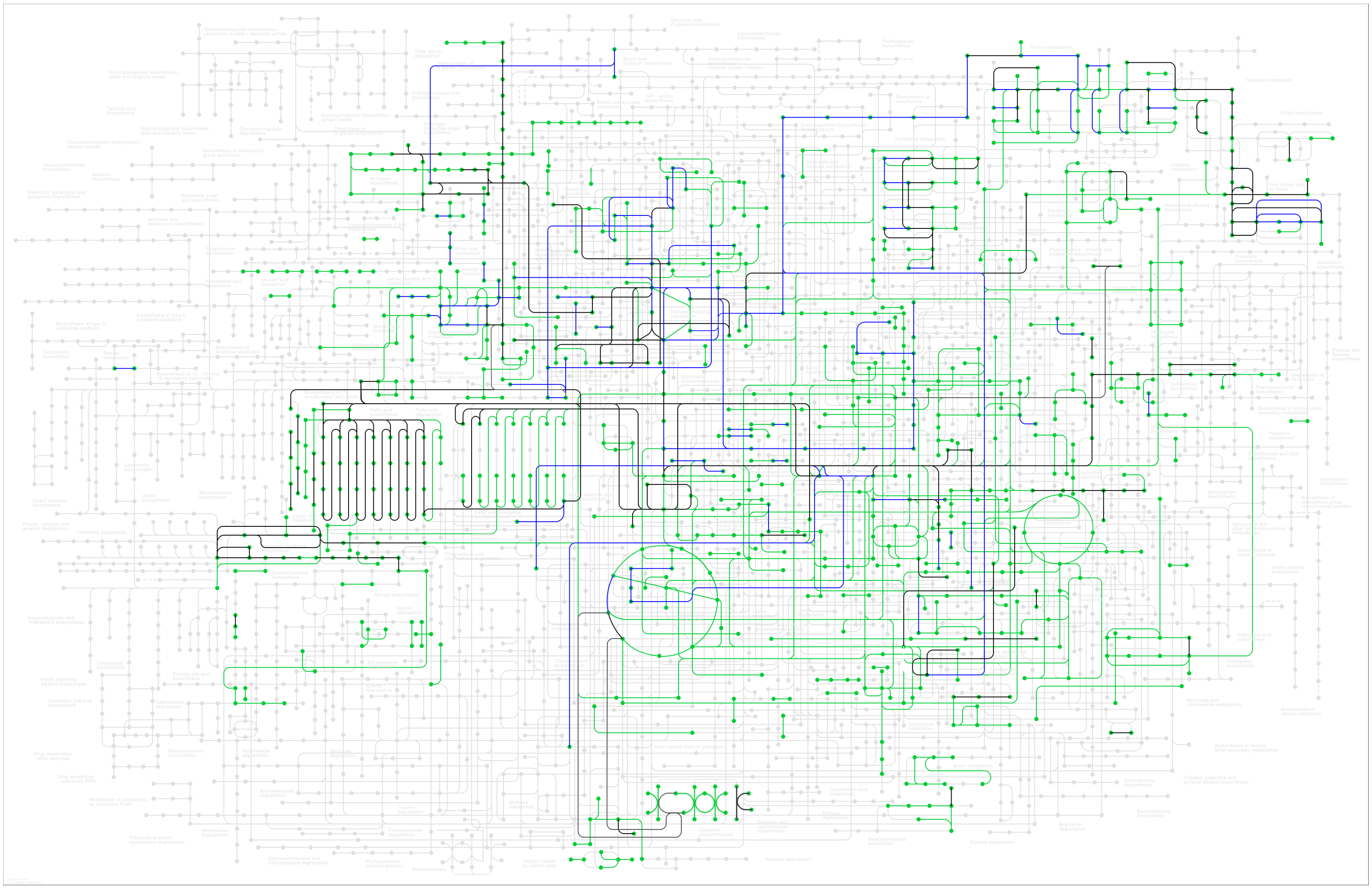

Network Topology

We have also used basic measures from the complex network theory to describe the MMNs (Fig.13-15) considered as a bipartite graph of reactions and metabolites; there are two different behaviours in the weight distributions for aerobic and anaerobic (Fig. 13), a pattern that is kept from the Wild Type networks, while the degree distributions are all similar (Fig. 14). The mean and standard deviation of the weights (Fig. 15) show how the aerobic networks in different conditions are divided in two sub-clusters.

Supplementary Figure 13. Average weight distribution in *MMN*s; aerobic and anaerobic networks have different distributions. Source data are provided as a Source Data file: SI Figs 13-15 Source Data.xlsx

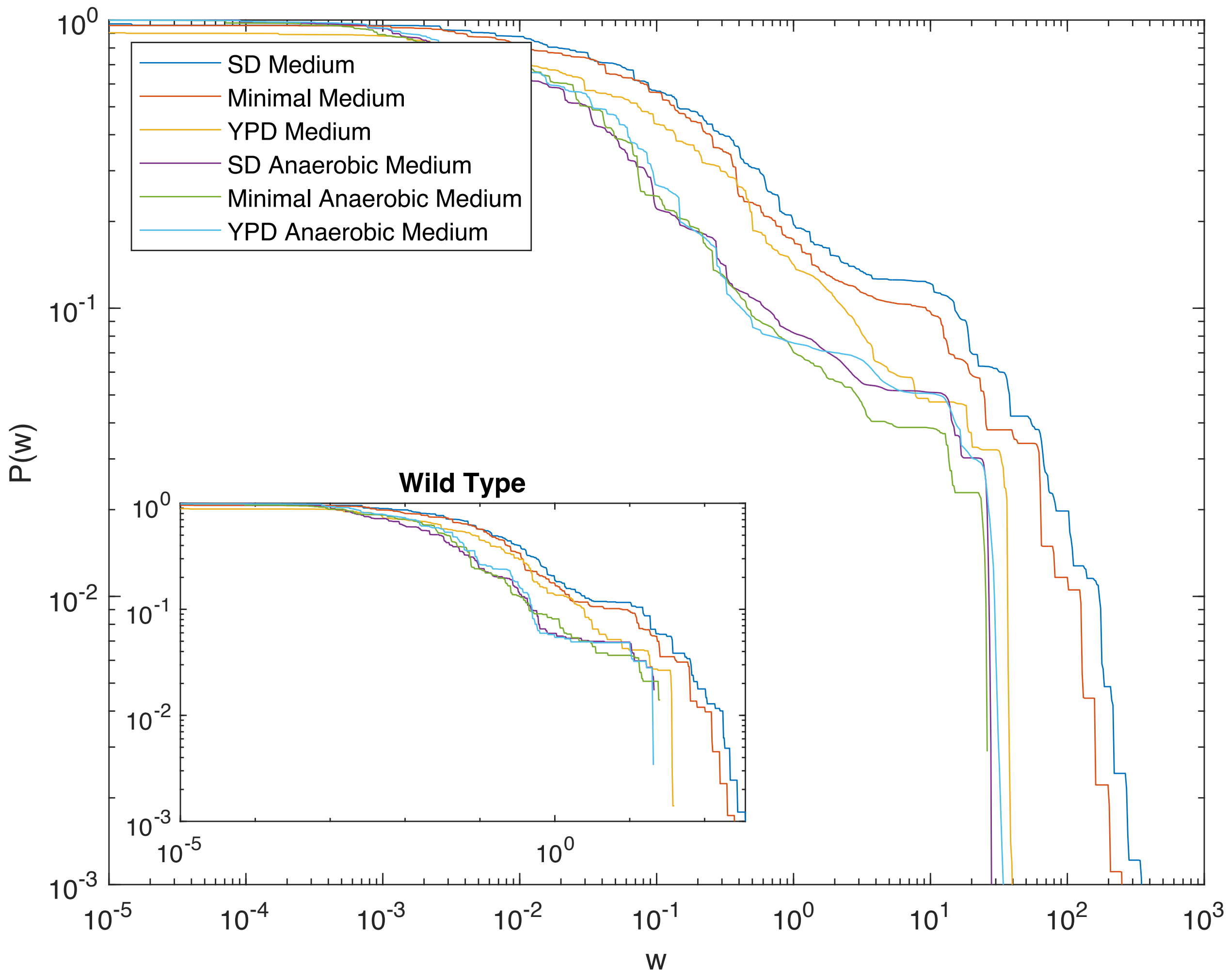

Supplementary Figure 14. Average degree distribution in *MMN*s, similar for all the 6 conditions. Source data are provided as a Source Data file: SI Figs 13-15 Source Data.xlsx

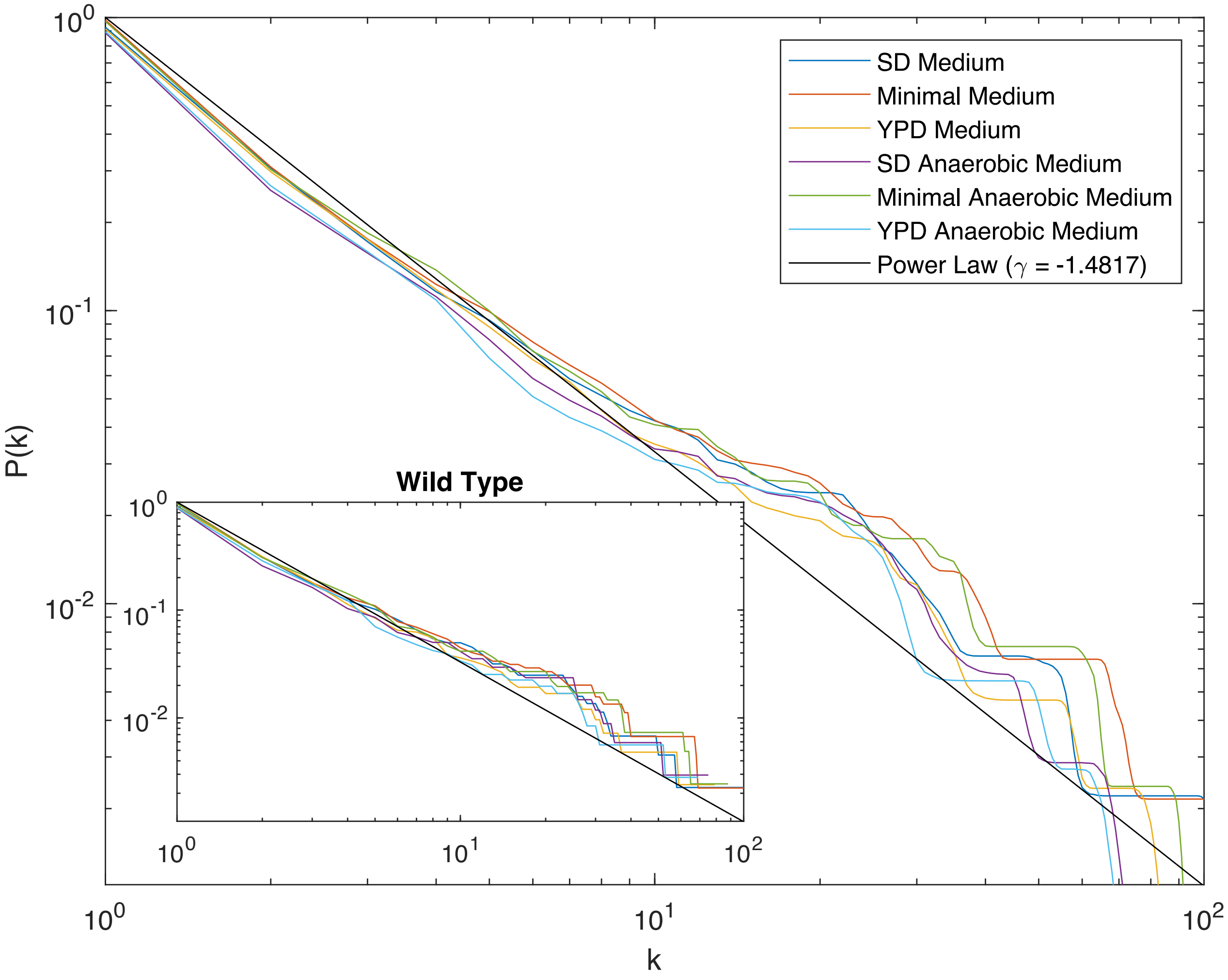

Supplementary Figure 15. Plot and frequency distribution of the mean and standard deviation of weights in the *MMN*s, divided in three operating regions. Source data are provided as a Source Data file: SI Figs 13-15 Source Data.xlsx

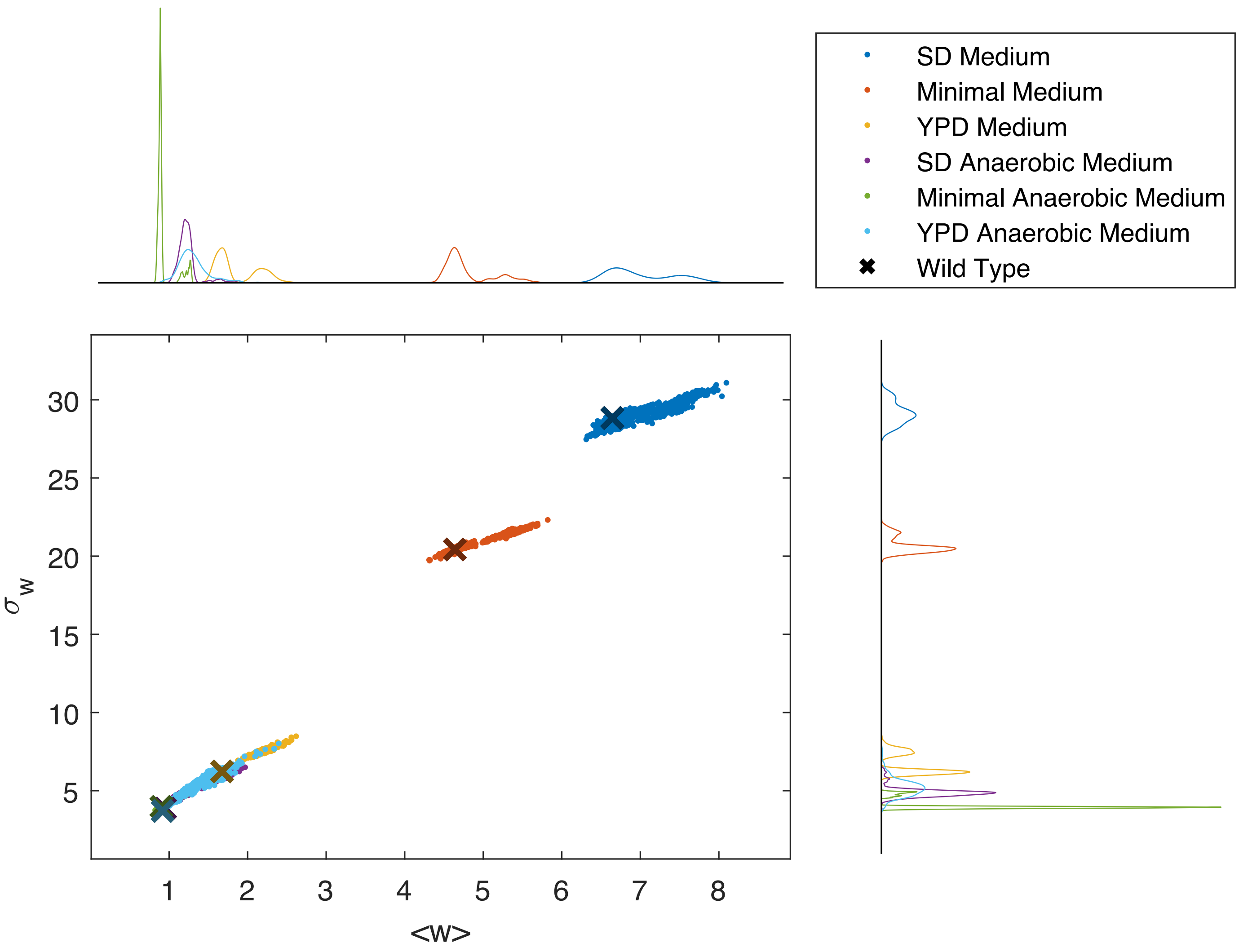

Algorithm 1 Minimal Media Algorithm

```
procedure Minimal Media(minBiomass, model, tol)
    Fluxes = getExchangeReaction(model)
    i = 0
    while i < tol do
        BackUp = Fluxes
        Fluxes = remove(rand(Fluxes))
        if FBA(model, Fluxes) >= minBiomass then
            i = 0
        else
            Fluxes = BackUp
            i = i + 1
            if i == tol then
                Fluxes = exhProc(Fluxes, model, minBiomass)
                if ¬(isempty(Fluxes)) then
                    i = 0
    return Fluxes
```

Algorithm 1 automatically defines a new minimal medium; it has three input parameters: *minBiomass* is the minimum value of the biomass function to be guaranteed, *model* is the given genome-scale metabolic model with the medium to be minimized, and *tol* is the number of attempts. The algorithm sequentially sets one of the exchange reaction bounds, chosen at random, to zero i.e., removing that compound from the simulated medium. The change is kept if the predicted biomass is still above the minimum, otherwise it is restored, and the procedure is repeated. If, after a number (*tol*) of attempts, an exchange reaction to be removed is not selected, a function implementing an exhaustive procedure is called. All the residual removals are tested in it and, if a feasible one is found, is returned to the main procedure; otherwise the procedure ends and returns an empty array, since no further exchange reactions bounds can be set to zero. The glucose, oxygen (if aerobic), and water exchange reactions bounds are fixed and not considered.

Supplementary Table 7. Media composition and bounds. The table shows the *Exchange Reactions* and the corresponding Bounds for the media used in this work. And empty entry in the table indicates that the given *Exchange Reaction* is not present in a given medium.

Exchange Reaction	Bounds					
	SD	Minimal	YPD	SD Anaerobic	Minimal An.	YPD An.
(R)-pantothenate exchange	-0.78		-0.001	-0.78		-0.001
4-aminobenzoate exchange	-0.78			-0.78		
adenine exchange	-0.78			-0.78		
ammonium exchange	-1000	-7.57	-1000	-1000	-1.66	-1000
biotin exchange	-0.78		-0.001	-0.78		-0.001
D-glucose exchange	-15	-15	-20	-15	-15	-20
folic acid exchange	-0.78			-0.78		
iron(2+) exchange	-1000	-0.01	-0.01	-1000		-0.01
L-alanine exchange	-0.1		-0.5	-0.1		-0.5
L-arginine exchange	-0.31		-0.5	-0.31		-0.5
L-asparagine exchange	-0.36		-0.5	-0.36		-0.5
L-aspartate exchange	-0.72		-0.5	-0.72		-0.5
L-cysteine exchange	-0.78		-0.5	-0.78		-0.5
L-glutamate exchange	-0.6		-0.5	-0.6		-0.5
L-glutamine exchange	-0.23			-0.23		
L-isoleucine exchange	-0.78			-0.78		
L-leucine exchange	-0.78		-0.5	-0.78		-0.5
L-lysine exchange	-0.78		-0.5	-0.78		-0.5
L-methionine exchange	-0.78		-0.5	-0.78		-0.5
L-phenylalanine exchange	-0.78			-0.78		
L-proline exchange	-0.78		-0.5	-0.78		-0.5
L-serine exchange	-0.47		-0.5	-0.47		-0.5
L-threonine exchange	-0.78		-0.5	-0.78		-0.5
L-tryptophan exchange	-0.78		-0.5	-0.78		-0.5
L-tyrosine exchange	-0.13		-0.5	-0.13		-0.5
L-valine exchange	-0.78			-0.78		
myo-inositol exchange	-0.78			-0.78		
nicotinate exchange	-0.78			-0.78		
oxygen exchange	-1000	-1000	-2			
phosphate exchange	-1000	-1.08	-1000	-1000	-0.24	-1000
potassium exchange	-1000			-1000		
pyridoxine exchange	-0.78			-0.78		
riboflavin exchange	-0.78		-0.001	-0.78		-0.001
sodium exchange	-1000			-1000		
sulphate exchange	-1000	-0.11	-1000	-1000	-0.03	-1000
thiamine(1+) exchange	-0.78		-0.001	-0.78		-0.001
uracil exchange	-0.78		-0.5	-0.78		-0.5
water exchange	-1000	-1000	-1000	-1000	-1000	-1000
choline exchange			-0.5			-0.5
deoxycytidine exchange			-0.5			-0.5
ergosterol exchange			-0.5	-0.01		-0.5
L-glycine exchange			-0.5			-0.5
guanine exchange			-0.5			-0.5
H+ exchange			-1000			-1000
L-histidine exchange			-0.5			-0.5
palmitate exchange			-0.5			-0.5
putrescine exchange			-0.001			-0.001
spermidine exchange			-0.001			-0.001
spermine exchange			-0.001			-0.001
stearate exchange			-0.5	-0.01		-0.5
thymidine exchange			-0.5			-0.5
myristate exchange			-0.5			-0.5
lanosterol exchange				-0.01	-0.01	-0.01
palmitoleate exchange				-0.05	-0.05	-0.05
zymosterol exchange				-0.01	-0.01	-0.01
14-demethyllanosterol exchange				-0.01	-0.01	-0.01
ergosta-5,7,22,24(28)-tetraen-3beta-ol exchange				-0.05	-0.05	-0.05
oleate exchange				-0.01	-0.01	-0.01

Algorithm 2 Minimization Algorithm

procedure MA(*pop*, *gen*, *model*, *minBiomass*) $P = initPop(pop)$
  for $i = 1 : gen$ **do**
    $P_t = geneticOperator(P_{i-1}, model, minBiomass)$ $P_t = sortPop(P_t \cup P_{i-1})$
    $P_t = P_t(1 : pop)$
    $P_i = aging(P_t, P_{i-1})$ $saveResult(P_i)$
  $R = loadResults()$
  $minSol = findMinimalSolutions(R)$
  return *minSol*

The Minimization Algorithm (MA) is the algorithm used to derive the Minimal Metabolic Networks (MMNs). MA iteratively improves the initial population (*pop*) for maximizing the number of knocked out genes (KO). Every element of the population represents a candidate solution (a strain), i.e. a set of genes that are knocked out. They are represented by a logical array, with every value corresponding to a gene in given genome-scale metabolic model (*model*). If the value is equal to 0 (false), the gene is still present in the model, otherwise it is knocked out. The initial population elements are all wild type strains, i.e. all-zeros arrays. The other two parameters of the algorithm are: *gen*, the maximum number of generations in the evolutionary cycle (in all simulations we used 5000 generations) and *minBiomass* is the minimum value of the biomass to be guaranteed.

Algorithm 3 Genetic Operator

```
function geneticOperator(P, model, minBiomass)
    for all p ∈P do
        ntrials = 0
        isFeasible = 0
        while ntrials < 10 and ¬isFeasible do
            p_t = selectNewRandKO(p)
            if (1 + FBA(p, model)/minBiomass) < 0.01 then
                p = p_t
                isFeasible = 1
            else
                ntrials = ntrials + 1
    return P
```

The Genetic Operator algorithm selects a new possible knockout over all the remaining active genes in the strain and it evaluates the new Biomass value. If the Biomass value satisfies the constraint (0.01, i.e. 1%, in the algorithm) the change is kept, and the new element-strain will enter in the new population (*P*), otherwise the searching is repeated till a feasible knockout is reached or a maximum number of trials (*10*) are performed.

Algorithm 4 Sorting Population Function

```
function sortPop(P ) KOs = getKOs(P ) i = 1
  while ∃p ∈P : no exists (p.front) do for all p ∈P do
        D_p = ManhattanDistance(p, P )
        if no exists (q ∈P : D_p(q) == 1 and KOs(q) == KOs(p) + 1 then
            p.front = i
        p.dist = sort(D_p, npoints)
        i = i + 1
  return P
```

The genetic operator constructs an offspring for every parent point in the population (*P*), and all these new points constitute the offspring population. The union of this and the parent population is then sorted using the Sorting Population Function. The idea of the sorting is that the points that were improved with a new knockout should be discarded. In order to do this the Hamming Distance of all the couples of points is evaluated.

Algorithm 5 Aging Function

```
function aging(P , Q, minBiomass)
  for all p ∈P do
        if ∃q ∈Q : q == p then
            p.age = q.age + 1
    else
          p.age = 0
          if 1/(1 + exp(10 − p.age/10)) < rand() then
              p = Backtracking(p, minBiomass)
  return P
```

All the points have a feature while in the population, their ages. The age can be defined as the numbers of the last generation in which the point has been present. If a point was not improved during the last generations of the algorithm, it is a candidate to be a minimal solution. If this is the case, there are no more knockouts that can be selected for the strain that satisfies the *minBiomass* constraint; there is no point then in keeping this point in the population. The age is then used as the variable of a sigmoid function representing the probability of a solution to be discarded from the population (*P*).
