# Supplementary material for: Minimisation of metabolic networks defines a new functional class of genes
Source: Nat Commun. 2024 Oct 31;15:9076. doi: 10.1038/s41467-024-52816-2 (PMC11528065; doi:10.1038/s41467-024-52816-2)
Supplement: Supplementary file 3 — Description of Additional Supplementary Files [file 41467_2024_52816_MOESM3_ESM.pdf]

Description of Additional Supplementary Files

File Name: Supplementary Data 1
Description: All results for all organisms studied. This table summarizes all the results in terms of frequency genes in the MMNs found by the algorithm Each sheet corresponds to a single organism's genome scale-model tested Frequency Definition For each organism, excluding S. cerevisiae, the first column represents the frequency in the MMNs found for that model for each of the genes in the second column. So, for example, a frequency of 1 means that the corresponding gene is present in the 100% of the MMNs, a frequency of 0.5 correspond to a gene present in the 50% of MMNs and so on. S. cerevisiae tabs. In these two tabs the first columns describe the genes, while there are more columns for the results, having considered more media. The 'All' columns refers to the frequency considering all the MMNs in all the media tested. The 'All Aerobic' columns refer to the frequency considering all the MMNs in the 3 aerobic media tested The 'All Anaerobic' columns refers to the frequency considering all the MMNs in the 3 anaerobic media tested Then there are the single columns referring to the MMNs found separately by the algorithm for each medium considered.

File Name: Supplementary Data 2
Description: We considered different metabolic networks resulting from their deletion and measured the reduced capability to produce precursors for the biomass pseudo-reaction, as defined in the metabolic network (by Flux Variability Analysis) detailed results in Supplementary Data 2. The table describes the simulated impact of the magnificent seven genes and all their possible combinations. For each deletion strain considered (columns) the ability of the metabolic network to grow or produce the maximum possible amount of a biomass' precursors (rows) is tested Global: the first two sheets consider an average of the strain in all the 6 media considered In the 'Perc' sheets are reported the percentual variation from the WT, colouring the strains with a more severe reduction The next tab report the absolute value of fluxes predicted All the next tabs consider a single medium (please consider that there is a duplicate for each tab).

File Name: Supplementary Data 3
Description: Information on the compartments and functions in the various used media. The table reports the frequency of KO divided by functionality or compartment location Each tab is for the function or compartment KO in the 6 different media. The columns B and C are the number of genes in WT and the relative weight in the genome scale model The columns D and E are similar, but referring to the smallest MMN found by the algorithm. The last two columns are again similar, but with the mean over all the MMNs found.

File Name: Supplementary Data 4
Description: Comparison with published prediction of dispensable and core essential genes. The table summarizes the comparison of our results with the study. The genes in the model marked as essential were not considered. In the first tab all the genes also present in the model are reported. The first 6 columns are taken from the reference study. In the columns labelled as 'Simulation (Presence in MMNs)' our results are reported instead. A scale of colour from green (or blue for dispensable) to red is used to highlight the genes with an outcome more or less similar to the result reference study. A last remark is sometimes

added in the last column. The next two tabs consider only the subsets of experimental results or the computer prediction of the reference study for the genes that are also present in the model The final tab report all the gene present in the reference study. Most of the genes are not present in the model, hence the data in our simulation for them are left blank.

File Name: Supplementary Data 5
Description: This supplementary data contains the list of genes considered as transporters in our study, with their SGD description and their category. This supplementary data lists all the transporter genes included in the WT metabolic model; it also provides information on their functional categories (e.g., amino acid transport, ammonium transport, ion transport, etc.) as well as the SGD description of their functional role (SGD accessed on 22.06.24).

File Name: Supplementary Data 6
Description: All primers used for construction of the deletion cassettes and those used for confirmatory PCRs.

File Name: Supplementary Data 7
Description: Growth rate, doubling times, and yields in both SD and YPD for the wild-type strains and the multiple mutants that we constructed.

File Name: Supplementary Data 8
Description: Statistical test used for comparison in Fig 2: two-sided Wilcoxon Test.
