# Supplementary material for: Minimisation of metabolic networks defines a new functional class of genes
Source: Nat Commun. 2024 Oct 31;15:9076. doi: 10.1038/s41467-024-52816-2 (PMC11528065; doi:10.1038/s41467-024-52816-2)
Supplement: Supplementary file 12 — Source Data [file 41467_2024_52816_MOESM12_ESM.zip › Source Data for the Figures 15 Sept/Fig 2 Statistical Data Boxplot.docx]

**Statistical test used for comparison in Fig 2: two-sided Wilcoxon Test**

- **Fig 2a**
  - **N = 10^4**
  - **Non-NED**
    - Minima: -0.04753
    - Maxima: -0.01667
    - centre (median): -0.02229
    - bounds of box (Q1 - Q3, i.e. 25th - 75th percentile): -0.02488, -0.02057
    - bounds of whiskers (Q1-1.5xIQR - Q3+1.5xIQR): -0.03133, -0.01667
  - **NED**
    - Minima: -0.05216
    - Maxima: -0.01814
    - centre (median): 0.02607
    - bounds of box (Q1 - Q3, i.e. 25th - 75th percentile): -0.02858, -0.02386
    - bounds of whiskers (Q1-1.5xIQR - Q3+1.5xIQR): -0.03565, -0.01814
- **Fig 2b**
  - **N = 10^3**
  - **Non-NED**
    - Minima: -0.09093
    - Maxima: 2.32386e-11
    - centre (median): -0.00577
    - bounds of box (Q1 - Q3, i.e. 25th - 75th percentile): -0.01086, -0.00341
    - bounds of whiskers (Q1-1.5xIQR - Q3+1.5xIQR): -0.02180, 2.32386e-11
    - percentile?
  - **NED**
    - Minima: -0.06589
    - Maxima: -0.00207
    - centre (median): -0.01562
    - bounds of box (Q1 - Q3, i.e. 25th - 75th percentile): -0.02060, -0.01056
    - bounds of whiskers (Q1-1.5xIQR - Q3+1.5xIQR): -0.03557, -0.00207
- **Fig 2c**
  - **N = 10^3**
  - **Non-NED**
    - Minima: -0.09602
    - Maxima: 2.32386e-11
    - centre (median): -0.00618
    - bounds of box (Q1 - Q3, i.e. 25th - 75th percentile): -0.01117, -0.00361
    - bounds of whiskers (Q1-1.5xIQR - Q3+1.5xIQR): -0.02235, 2.32386e-11
  - **NED**
    - Minima: -0.11415
    - Maxima: -0.01900
    - centre (median): -0.03392
    - bounds of box (Q1 - Q3, i.e. 25th - 75th percentile): -0.03569, -0.02959
    - bounds of whiskers (Q1-1.5xIQR - Q3+1.5xIQR): -0.04449, -0.02055
